# Study on Compression Properties and Construction Applications of Loess Filling Materials for High Embankments Along G85 Expressway in Eastern Gansu Province

**DOI:** 10.3390/ma18163811

**Published:** 2025-08-14

**Authors:** Wei Sun, Yongle Chen, Xiaoli Yi, Jinpeng Zhao, Lulu Liu, Hongli Wang, Meng Han

**Affiliations:** 1 China Railway First Group Co., Ltd., Xi’an 712000, China; 18301970397@163.com (W.S.); bananatiny@163.com (Y.C.); zhao19980522@126.com (X.Y.); 2 School of Civil Engineering, Dalian University of Technology, Dalian 116024, China; peng19_98@163.com (J.Z.); hanmeng320@mail.dlut.edu.cn (M.H.); 3 School of Highway, Chang’an University, Xi’an 710064, China; 4 School of Mechanics and Civil Engineering, China University of Mining and Technology, Xuzhou 221116, China; 02224711@cumt.edu.cn

**Keywords:** subgrade engineering, loess, compaction degree, porosity ratio, compression coefficient

## Abstract

Based on the G85 high-fill subgrade project in east Gansu Province, this study conducts one-dimensional compression tests in the laboratory on both disturbed and in situ-compacted loess. Through the combination of the test results of remolded soil, compaction standards for each layer of the subgrade fill are established, and quality inspections of the compacted subgrade are performed. The experimental results demonstrate that the compression deformation of remolded loess exhibits a positive correlation with compaction degree and a negative correlation with moisture content. Under constant compaction degree conditions, axial pressure and deformation follow a linear relationship, whereas under fixed conditions, the relationship adheres to a quadratic trend. Specimen void ratios show minimal variation within the 25–100 kPa stress range but undergo significant reduction between 100 and 400 kPa. Under an axial compressive load of 100–200 kPa, the compression coefficient at a height of 10 m within the subgrade ranges from 0.163 to 0.171 MPa^−1^. At a height of 6 m, it ranges from 0.177 to 0.183 MPa^−1^, and at 1 m, from 0.183 to 0.186 MPa^−1^. These values indicate that the compaction quality throughout the subgrade corresponds to a low compressibility level. However, the compaction quality near the slopes on both sides is slightly lower than that along the centerline of the subgrade. Overall, the compaction quality meets the required standards.

## 1. Introduction

Loess is a regionally specific soil widely distributed across the northwestern region of China. It typically forms in arid environments and covers an area of approximately 540,000 km^2^. With the strategic implementation of the Belt and Road Initiative, urban development and road maintenance in western China have experienced unprecedented growth [1,2,3,4]. Due to the limited variety of available fill materials in the northwest region, at the same time, to reduce construction costs while efficiently utilizing local resources, the principle of “using local materials for local filling” was adopted during road construction. As a result, loess was extensively used as a fill material in the construction of subgrades [5,6,7,8]. Consequently, the study of key properties of loess fill materials, such as compressibility, stress behavior, and microscopic mechanisms, has attracted considerable attention from researchers in the field of geotechnical engineering [9,10,11].

Influenced by the topography and geological conditions of the northwest region, high-fill and cut–fill subgrades have become common cross-sectional forms in highway construction [12,13,14]. However, settlement deformation caused by both the intrinsic properties of the fill material and construction techniques poses significant safety risks to highway operation. To address these hazards, numerous domestic and international scholars have conducted extensive research on the compression characteristics of fill materials [15,16,17]. Jiang et al. [18] proposed a discrete element method (DEM) to investigate the macroscopic and microscopic mechanical behavior of loess fill under one-dimensional compression. A bonding contact model for loess was developed by incorporating the relationship between bonding strength and initial water content within the DEM framework. Qin et al. [19] examined the one-dimensional compressibility and creep behavior of unsaturated compacted loess using both the incremental loading method and the constant strain rate method. Ge et al. [20] analyzed the compressibility and creep behavior of compacted loess. They derived key parameters including the primary compression index, secondary compression index, and moisture content. One-dimensional compression tests, combined with mercury intrusion porosimetry and scanning electron microscopy (SEM), were used to examine the collapse behavior of loess under axial loading in a specific area of Xi’an under varying compaction conditions. Guo et al. [21] enhanced the compressibility characteristics of loess fill by injecting novel polymers. One-dimensional compression tests were conducted to evaluate the compressibility of both solidified and untreated loess under varying water contents. To investigate the mechanical behavior of unsaturated loess during humidification, Xing et al. [22] focused on loess from Yili, Xinjiang. Using a self-developed water absorption meter, they measured the static earth pressure coefficient and matric suction during the humidification process. The variation trends of soil deformation, the static earth pressure coefficient, and suction with respect to moisture content were analyzed.

Located in the western Loess Plateau of China, the eastern Gansu Province region exhibits loess characterized by widespread distribution, significant thickness, and pronounced collapsibility. These properties display marked regional heterogeneity, posing substantial challenges to highway route design, on-site construction, and long-term maintenance management [23,24,25]. The strength and long-term service performance of loess subgrades are critically influenced by the intrinsic properties of the loess material itself, road construction quality, and prevailing environmental conditions. Consequently, highways constructed in this region are particularly susceptible to collapsibility-induced distress, manifesting as differential settlement of the subgrade, slope spalling, scouring, and collapse [26]. These prevalent geotechnical issues severely compromise traffic safety, highway capacity, and service levels.

**Figure 1:**
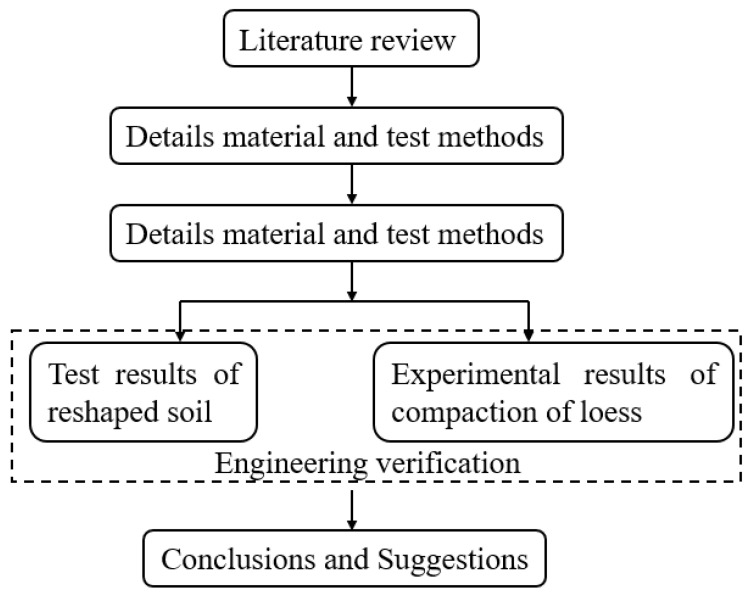
The flowchart.

Based on a high-fill subgrade project in eastern Gansu Province, this study conducts laboratory one-dimensional compression tests on both disturbed and in situ-compacted loess. Based on the compression test results of remolded soil, compaction standards for each layer of the fill subgrade are established, and the compaction quality of the subgrade is evaluated. The findings offer academic guidance for the construction of high-fill subgrades and contribute to disaster prevention and mitigation in loess regions. Figure 1 is the flowchart of this study.

## 2. Test Scheme

### 2.1. Site Conditions

**Figure 2:**
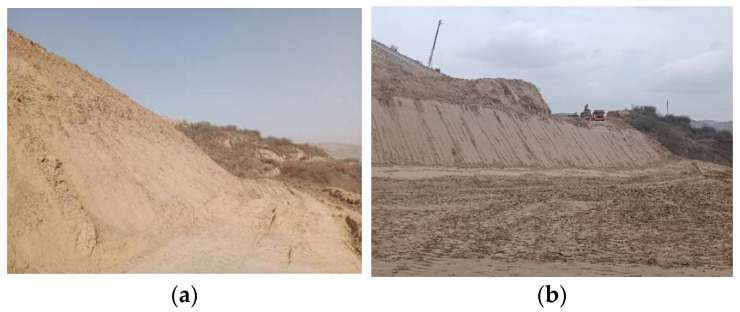
Field conditions. (**a**) Soil sampling location; (**b**) subgrade cross-section.

**Figure 3:**
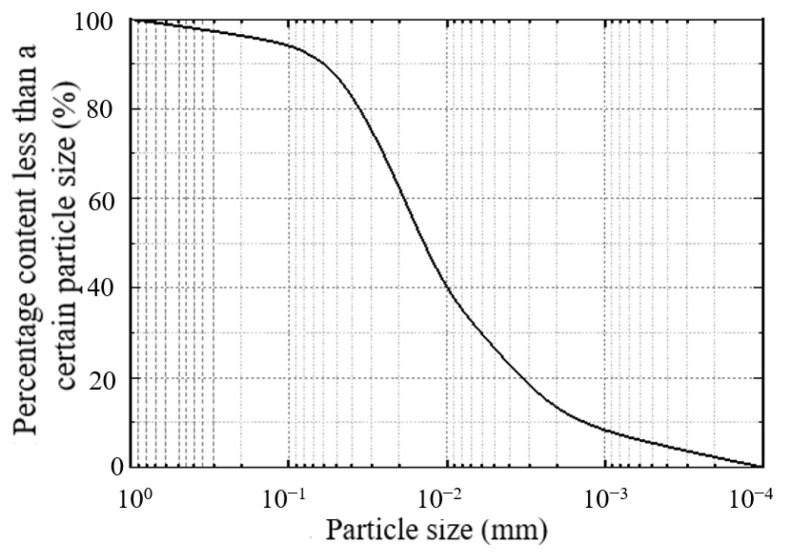
The grain size distribution.

The soil samples were taken from a certain fill section in Yuanzhou District, Guyuan City, Ningxia Province. The soil sampling site is shown in Figure 2, and the grain size distribution is shown in Figure 3. The basic physical and mechanical indicators of the unformed loess measured are shown in Table 1.

### 2.2. Remolded Soil Test

**Figure 4:**
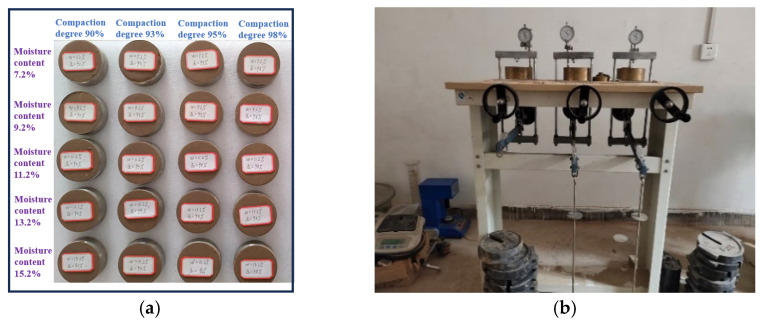
Soil samples and consolidation apparatus. (**a**) Remolded loess; (**b**) WG-type single-lever consolidometer.

After the original loess on site was crushed and dried, a total of 40 reconstituted soil samples with different compaction degrees (*K* = 90%, 93%, 95%, 98%) and different moisture contents (*w* = 7.2%, 9.2%, 11.2%, 13.2%, 15.2%) were made (including a set of control experiments), and circumferential knife samples with a size of 60 cm^3^ were produced. A WG-type single-lever consolidation instrument was used for the tests, with applied pressure levels of 25 kPa, 50 kPa, 100 kPa, 200 kPa, 300 kPa, and 400 kPa. The detailed test plan is presented in Table 2. The reconstituted soil sample and the WG-type consolidation apparatus are shown in Figure 4.

## 3. Results and Analysis

In this study, one-dimensional compression tests were conducted on reshaped soil samples with different compaction degrees and moisture contents to investigate the compression deformation characteristics of reshaped loess, providing academic guidance for the construction of high-fill subgrades in loess areas. The research contents include the relationship between the deformation and pore ratio of each soil sample and the axial pressure, as well as the analysis of the compressibility index of each soil sample.

### 3.1. Analysis of Axial Compression and Deformation of Remolded Soil

**Figure 5:**
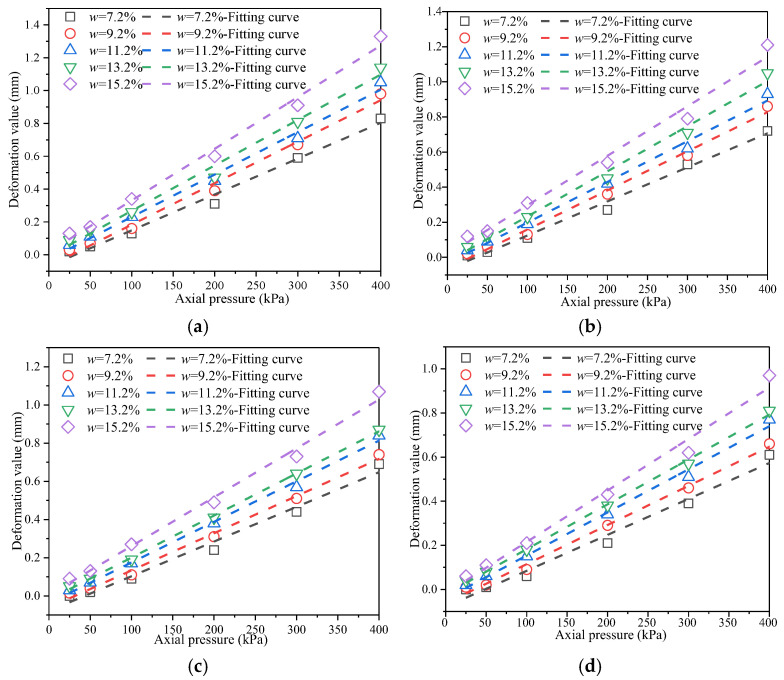
*p*-*s* curves of remolded soil under different compaction degrees. (**a**) Compaction degree *K* = 90%; (**b**) compaction degree *K* = 93%; (**c**) compaction degree *K* = 95%; (**d**) compaction degree *K* = 98%.

Figure 5 presents the *p*-*s* curves of remolded soil samples with varying compaction degrees. According to the analysis of the test results, as the axial pressure increases, the axial deformation of each sample exhibits a clear upward trend. For samples with identical compaction degrees but varying moisture contents, axial pressure and deformation exhibit a linear relationship. In terms of the influence of moisture content, when the axial pressure *P* < 100 kPa, soil samples with a moisture content *w* < 11.2% generally exhibit relatively small deformation; in contrast, samples with a moisture content *w* > 13.2% exhibit significant deformation even under an axial pressure as low as 50 kPa. This phenomenon indicates that moisture content has a substantial impact on the mechanical properties of the soil, with an excessively high moisture content significantly reducing the soil’s resistance to deformation. The test data further show that under the condition of a fixed compaction degree, for every 2% increase in moisture content, the average deformation under the same axial pressure increases by approximately 15%. In terms of the influence of compaction degree, the research results clearly indicate that there is a significant negative correlation between the compaction degree of soil samples and the deformation. When the compaction degree *K* < 93%, soil samples at all moisture content levels exhibit large deformations. The order of deformation magnitude is as follows: *S*_(*w*=7.2%)_ < *S*_(*w*=9.2%)_ < *S*_(*w*=11.2%)_ < *S*_(*w*=13.2%)_ < *S*_(*w*=15.2%)_. Especially under the working condition with a compaction degree of 90%, when the axial compressive load increases from 50 kPa to 150 kPa, the deformation increases by approximately 120%. This fully indicates that the stability of low-pressure-compacted soil under load is relatively poor; in contrast, when the compaction degree is increased to over 95%, the deformation of each soil sample under the same load conditions can be reduced by 40–50%, and the deformation control effect is remarkable. Based on the above research findings, it is recommended that a stratified compaction standard be adopted for the construction of first-class highways. For lower subgrades, which bear relatively low loads, the compaction degree should not be less than 90%. For the middle subgrade, the primary load-bearing layer, the compaction degree should be maintained above 93%. For the upper subgrade, which directly bears traffic loads, it is essential to ensure that the compaction degree exceeds 95%. Additionally, during construction, the moisture content of the fill material should be strictly controlled within ±2% of the optimal moisture content to achieve optimal compaction and ensure subgrade stability [27,28].

**Figure 6:**
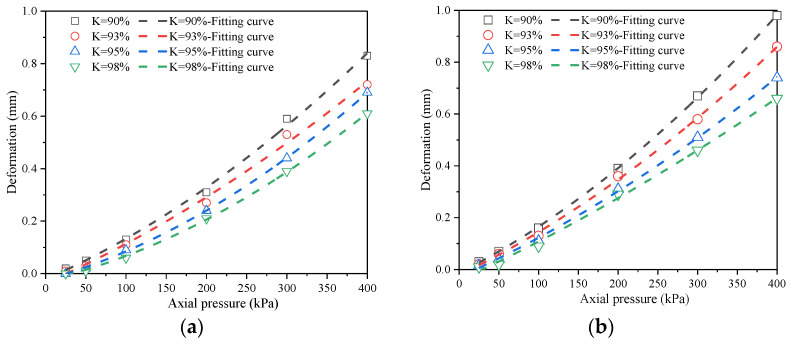

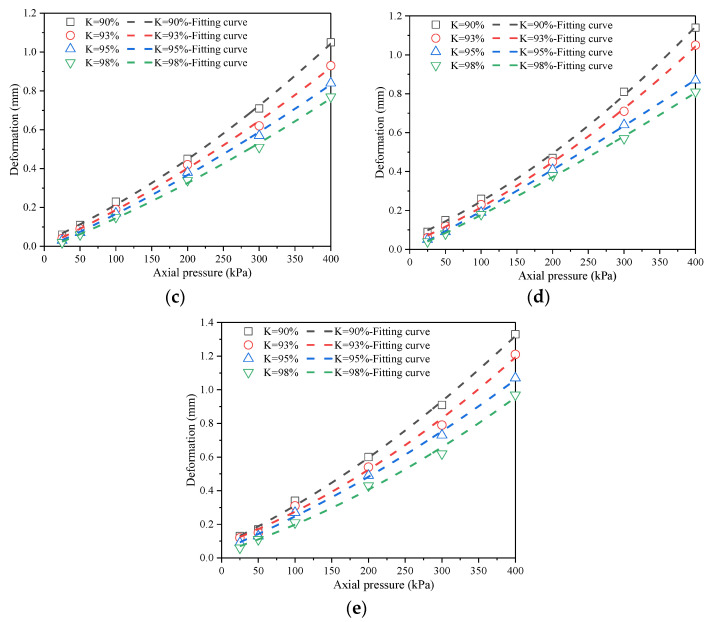
*p*-*s* curves of remolded soil under different moisture contents. (**a**) Moisture content *w* = 7.2%; (**b**) moisture content *w* = 9.2%; (**c**) moisture content *w* = 11.2%; (**d**) moisture content *w* = 13.2%; (**e**) moisture content *w* = 15.2%.

Figure 6 presents the *p*-*s* curves of remolded soils with varying moisture contents.

As shown in Figure 6, the deformation of each soil sample increases with axial pressure, and the rate of deformation growth accelerates progressively. For soil samples with the same moisture content but different compaction degrees, the relationship between axial pressure and deformation follows a quadratic trend. When the axial pressure *P* < 100 kPa, the deformation of each specimen remains below 0.3 mm. As axial pressure increases, the differences in deformation among soil samples with varying moisture contents and compaction degrees become more pronounced. Under a fixed compaction degree, higher moisture content results in greater deformation under the same axial load [29,30]. This indicates a positive correlation between moisture content and deformation. When the compaction degree exceeds 95%, the deformation of all soil samples remains relatively small.

### 3.2. Analysis of Axial Pressure and Pore Ratio of Reshaped Soil

**Figure 7:**
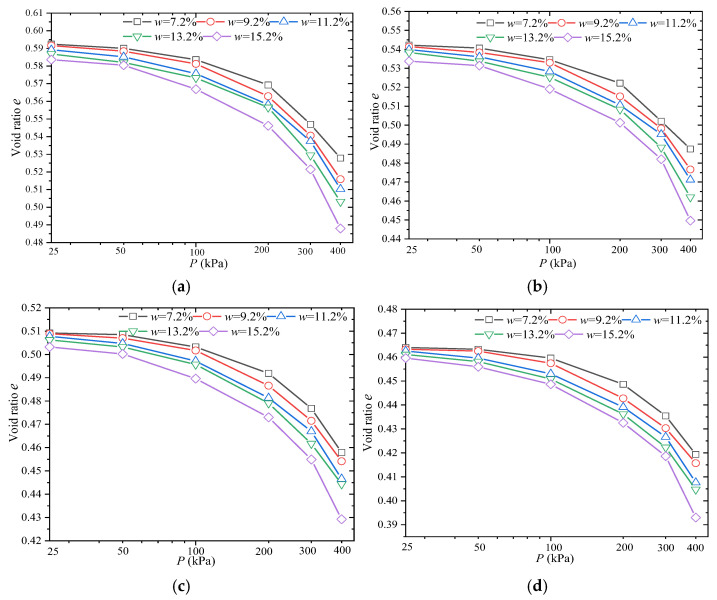
*e*-*p* curves of remolded soil samples. (**a**) Compaction degree *K* = 90%; (**b**) compaction degree *K* = 93%; (**c**) compaction degree *K* = 95%; (**d**) compaction degree *K* = 98%.

Figure 7 shows the relationship between the axial pressure of the soil sample and the pore ratio with the variation in moisture content under different compaction degrees.

It can be seen from Figure 7 that with the increase in axial pressure, the pore ratio e of each soil sample shows a decreasing trend, and the trend gradually intensifies after slow changes. The pore ratio change in the soil sample at an axial pressure of 25–200 kPa is relatively small, while the pore ratio change at 200–400 kPa is relatively large. When the axial pressure *P* < 100 kPa and the moisture content *w* < 11.2%, the pore ratio of each soil sample remains relatively stable. However, when *P* > 100 kPa and *w* > 11.2%, the decrease in the pore ratio becomes more pronounced. At a fixed moisture content, an increase in compaction degree results in a smaller change in the pore ratio under the same axial pressure.

These findings indicate that the compaction degree of a soil sample is inversely proportional to its pore ratio. When the compaction degree *K* < 93%, soil samples across all moisture content levels exhibit significant variation in the pore ratio, specifically following the order *e*_(*w*=7.2%)_ < *e*_(*w*=9.2%)_ < *e*_(*w*=11.2%)_ < *e*_(*w*=13.2%)_ < *e*_(*w*=15.2%)_. This trend is particularly evident when the compaction degree *K* = 90%, where the reduction in the pore ratio becomes most pronounced as axial compressive load increases. In contrast, when the compaction degree *K* > 95%, changes in the pore ratio of each soil sample are minimal.

### 3.3. Analysis of Compressibility of Remolded Soil

In this section, the *e*-*p* curves of the soil samples are further analyzed to determine the compression coefficient, compression modulus, and compression index of each sample. The derivation formulas for these parameters are provided in Equations (1)–(3).

Compression coefficient:

(1) $$a=\frac{\Delta e}{\Delta p}=\frac{e_{1}-e_{2}}{p_{2}-p_{1}}$$

Compression modulus:

(2) $$E_{s}=\frac{\Delta p}{\frac{\Delta H}{H_{1}}}=\frac{\Delta p}{\frac{\Delta e}{1+e_{1}}}=\frac{1+e_{1}}{a}$$

Compression index:

(3) $$C_{c}=\frac{e_{1}-e_{2}}{lgp_{2}-lgp_{1}}$$

Note: a—compression coefficient; Δe—pore ratio difference; $e_{1}$—initial void ratio; $e_{2}$—final void ratio; $p_{1}$—load (100 kPa); $p_{2}$—load (200 kPa); Δp—load difference; $E_{s}$—compression modulus; ΔH—compression deformation value; $H_{1}$—initial specimen height; and $C_{c}$—compression index.

#### 3.3.1. Compression Coefficient

**Figure 8:**
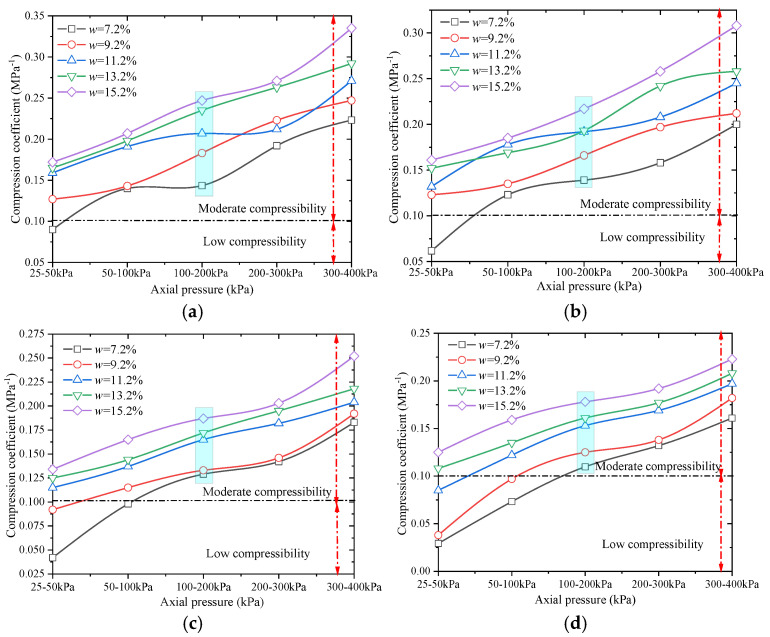
Variation in compression coefficient for remolded soil samples with axial pressure. (**a**) Compaction degree *K* = 90%; (**b**) compaction degree *K* = 93%; (**c**) compaction degree *K* = 95%; (**d**) compaction degree *K* = 98%.

Figure 8 shows the variation in the measured compression coefficient with axial compressive load for different compaction degrees and moisture contents.

As shown in Figure 6, the compression coefficient of remolded loess is lowest within the axial pressure range of 25–50 kPa and highest within the range of 300–400 kPa across all compaction degrees. At a fixed compaction degree, the soil sample with a moisture content of 7.2% exhibits the smallest compression coefficient. Similarly, at a fixed moisture content, the sample with a compaction degree of 98% has the smallest compression coefficient. These results indicate that the compressibility of a soil sample is positively correlated with moisture content and negatively correlated with compaction degree [31]. When the axial pressures *P*_1_ = 100 kPa and *P*_2_ = 200 kPa are applied, the compression coefficient of the remolded soil sample with a compaction degree of 90% ranges from 0.1435 to 0.247 MPa^−1^. For compaction degrees of 93%, 95%, and 98%, the corresponding ranges are 0.139–0.217 MPa^−1^, 0.129–0.187 MPa^−1^, and 0.1098–0.178 MPa^−1^, respectively. All remolded soil samples fall within the range of low compressibility.

#### 3.3.2. Compression Modulus

**Figure 9:**
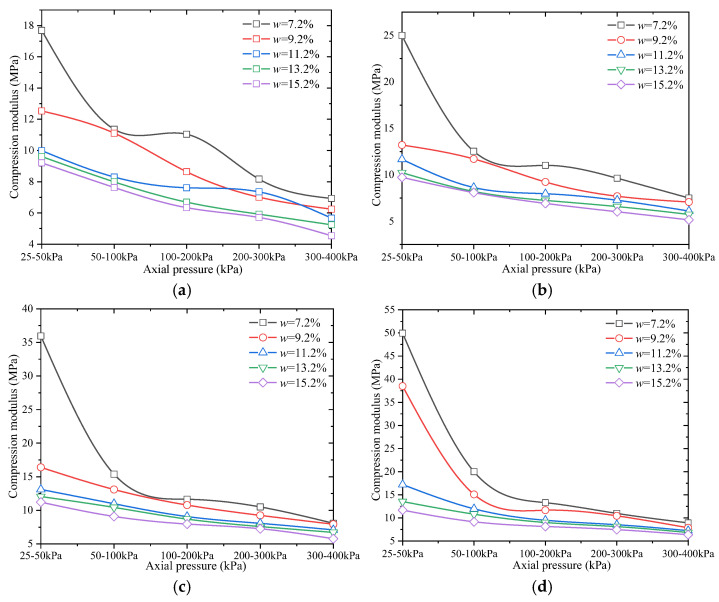
Variation in compression modulus of remolded soil samples with axial pressure. (**a**) Compaction degree *K* = 90%; (**b**) compaction degree *K* = 93%; (**c**) compaction degree *K* = 95%; (**d**) compaction degree *K* = 98%.

Through further analysis of the *e*-*p* curves in conjunction with Figure 6, the variation in the compression modulus for each soil sample under different axial pressures was determined. The results are presented in Figure 9.

As shown in Figure 9, under the same compaction degree, the compression modulus of the fill material decreases with increasing moisture content. Conversely, under the same moisture content, the compression modulus increases as the compaction degree increases. Therefore, the compression modulus is inversely proportional to moisture content and directly proportional to compaction degree. When the applied loads are *P*_1_ = 100 kPa and *P*_2_ = 200 kPa, the compression modulus of remolded soil samples varies as follows: for a compaction degree of 90%, the range is 6.34–11.04 MPa; for 93%, it is 6.92–11.02 MPa; for 95%, it is 7.96–11.65 MPa; and for 98%, it is 8.14–13.29 MPa.

#### 3.3.3. Compression Index

**Figure 10:**
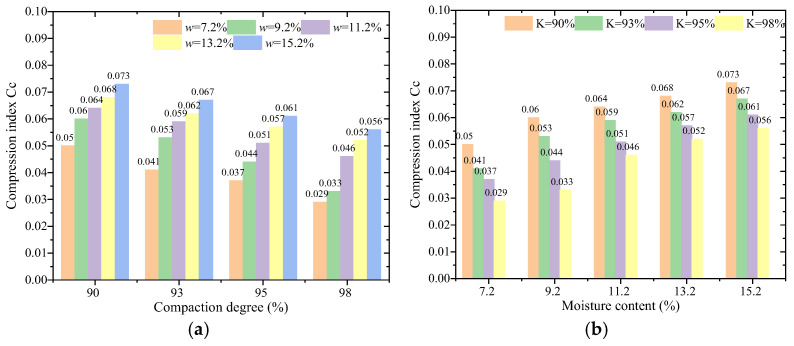
Compression index of remolded soil samples. (**a**) Different compaction degrees; (**b**) different moisture contents.

Through further analysis of the *e*-*p* curves, in combination with the variation trends of the compression coefficient and compression modulus, the compression index of each remolded loess sample was determined under the applied loads *P*_1_ = 100 kPa and *P*_2_ = 200 kPa. The results are presented in Figure 10.

As shown in Figure 10, the compression index of each remolded soil sample remains below 0.1 within the compaction degree range of 90%–98% and the moisture content range of 7.2%–15.2%. At a fixed compaction degree, the compression index increases with increasing moisture content. Conversely, at a fixed moisture content, the compression index decreases as the compaction degree increases. Overall, the compression index is positively correlated with moisture content and negatively correlated with compaction degree.

## 4. Engineering Examples

Based on the results of the indoor one-dimensional compression test, in conjunction with on-site compaction standards for high-fill subgrade sections, the compaction quality of the project section was evaluated. According to field specifications, the required compaction degrees are as follows: not less than 90% for the 0–2 m layer, not less than 93% for the 2–6 m layer, and not less than 95% for the 6–12 m layer. On site, the moisture content of each compacted layer is controlled according to the optimal moisture content of 11.2%.

According to the one-dimensional compression test results of remolded soil, under the optimal moisture content, the compression coefficients corresponding to compaction degrees of 90%, 93%, 95%, and 98% under an axial pressure of 100–200 kPa are 0.207 MPa^−1^, 0.192 MPa^−1^, 0.165 MPa^−1^, and 0.144 MPa^−1^, respectively.

### 4.1. The Relationship Between Axial Pressure and Deformation of Compacted Loess in the Subgrade

**Figure 11:**
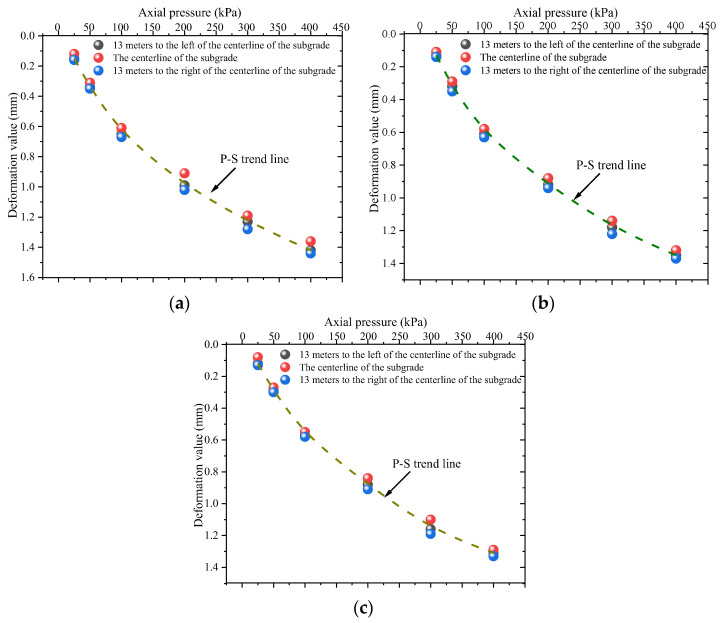
*p*-*s* curves of compacted loess: (**a**) 1 m subgrade layer (compaction degree *K* = 90%); (**b**) 6 m subgrade layer (compaction degree *K* = 93%); (**c**) 10 m subgrade layer (compaction degree *K* = 98%).

Figure 11 presents the *p*-*s* curves of compacted loess at depths of 1 m, 6 m, and 10 m within the subgrade.

As shown in Figure 11, the vertical compression deformation of the specimens increases with increasing axial pressure, following a trend that approximates an inverse proportional function. The settlement at each depth along the subgrade centerline is slightly smaller than that observed on the left and right sides. This suggests that, during the compaction process, the compaction quality at the center of the subgrade is significantly higher than near the side slopes. At the same time, both side slopes, particularly at the toe and shoulder areas, are susceptible to weathering cycles and precipitation infiltration. This leads to amplified subgrade deformation and can induce localized cracking. Consequently, engineering practice necessitates enhanced drainage and reinforcement measures for fill slopes.

Under axial pressure ranging from 0 to 400 kPa, the vertical deformation of compacted loess is approximately 1.40 mm at the 1 m subgrade level, 1.38 mm at the 6 m level, and 1.35 mm at the 10 m level.

### 4.2. Relationship Between Porosity Ratio of Compacted Loess and Load

**Figure 12:**
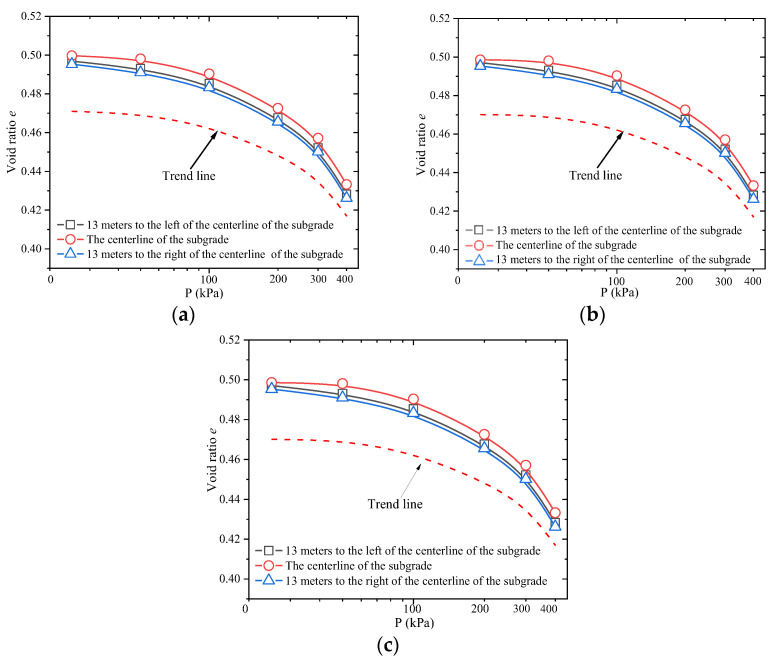
*e*-*p* curves of compacted loess: (**a**) 1 m subgrade layer (compaction degree *K* = 90%); (**b**) 6 m subgrade layer (compaction degree *K* = 93%); (**c**) 10 m subgrade layer (compaction degree *K* = 98%).

The *e*-*p* curves of each compacted loess were obtained through further processing of the *p*-s curves, as shown in Figure 12.

As shown in Figure 12, the pore ratio of the samples decreases with increasing axial compressive load. The pore ratio of compacted loess at the centerline of each subgrade level is slightly higher than that at the sides, further indicating that the compaction quality near the slopes is relatively low. To address this, dynamic compaction operations should be enhanced during construction. In the later stages, slope stabilization and drainage measures should be strengthened to prevent slope failure and subgrade settlement caused by heavy rainfall.

### 4.3. The Compression Coefficient of Compacted Loess in the Subgrade

**Figure 13:**
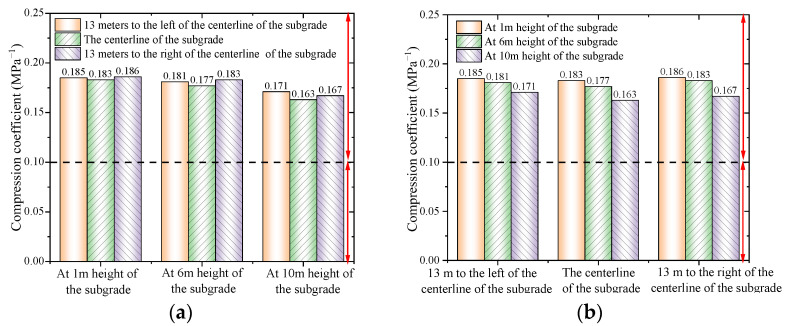
Compression coefficients of compacted loess. (**a**) At different embankment layers; (**b**) at different locations.

The compression coefficients of each soil sample at *P*_1_ = 100 kPa and *P*_2_ = 200 kPa were obtained by further processing the data, combining the *p*-*s* curves of compacted loess, as shown in Figure 13.

It can be known from Figure 13 that at a height of 1 m of the subgrade, 13 m to the left of the subgrade centerline, and 13 m to the right of the subgrade centerline, the compression coefficients under an axial pressure of 100–200 kPa are 0.185 MPa^−1^, 0.183 MPa^−1^, and 0.186 MPa^−1^, respectively. At a height of 6 m of the subgrade, 13 m to the left of the subgrade centerline, and 13 m to the right of the subgrade centerline, the compression coefficients under an axial pressure of 100–200 kPa are 0.181 MPa^−1^, 0.177 MPa^−1^, and 0.183 MPa^−1^, respectively. At a height of 10 m of the subgrade, 13 m to the left of the subgrade centerline, and 13 m to the right of the subgrade centerline, the compression coefficients under an axial pressure of 100–200 kPa are 0.171 MPa^−1^, 0.163 MPa^−1^, and 0.167 MPa^−1^, respectively. Each layer of the fill section shows that the compaction degree near the slope is slightly less than that at the centerline of the subgrade.

According to subgrade compaction standards, the required compaction degree is no less than 90% for the 0–2 m layer, no less than 93% for the 2–6 m layer, and no less than 95% for the 6–12 m layer. Based on the results of the one-dimensional compression test of remolded soil, it can be concluded that the compaction degree at a height of 1 m meets the standard of no less than 90%, the compaction at 6 m meets the requirement of no less than 93%, and the compaction at 10 m satisfies the requirement of no less than 95%.

Analysis demonstrates that soil compressibility exhibits a positive correlation with compaction degree but a negative correlation with moisture content. Uniaxial compression tests on remolded loess further suggest that subgrade compaction should be executed at the optimum moisture content, with the degree of compaction maintained at or above 93% for the lower subgrade and 96% for the upper subgrade [32]. Additionally, compaction efforts require intensification near slope interfaces across all structural layers. Concurrently, slope reinforcement and drainage measures must be enhanced to mitigate adverse impacts of precipitation and environmental hygrothermal variations on soil strength and deformation characteristics [33,34,35,36].

## 5. Conclusions

In this study, indoor one-dimensional compression tests were conducted on remolded soil and compacted loess from a fill embankment located in eastern Gansu Province. The compression deformation characteristics of remolded loess under varying compaction degrees and moisture contents, as well as those of compacted loess at different embankment layers, were analyzed. Based on these findings, the compaction quality of the fill embankment was evaluated. The main conclusions are as follows:

(1) Under the same compaction degree, the axial pressure of and variation in soil samples with different moisture contents form a linear function variation relationship; under the same moisture content, the axial pressure of and variation in soil samples with different compaction degrees form a quadratic function variation relationship.
(2) With the increase in axial pressure, the pore ratio of each specimen gradually decreases. The pore ratio varies slightly within the load range of 25–100 kPa and significantly within the range of 100–400 kPa. The change in the pore ratio is negatively correlated with the compaction degree of the sample and positively correlated with the moisture content. Compression deformation is positively correlated with the compaction degree of the sample and negatively correlated with the moisture content.
(3) Under an axial pressure of 100–200 kPa, the compression coefficient at a height of 10 m in the embankment ranges from 0.163 to 0.171 MPa^−1^; at 6 m, it ranges from 0.177 to 0.183 MPa^−1^; and at 1 m, it ranges from 0.183 to 0.186 MPa^−1^. These values indicate that the compacted loess across all embankment layers is characterized by low compressibility.
(4) A comparison between the one-dimensional compression test results of remolded soil and those of compacted loess at various embankment levels confirms that the overall compaction meets the specified standards. However, the compaction quality near the side slopes of each layer is relatively lower, and additional dynamic compaction measures are recommended to improve uniformity.

## Figures and Tables

**Table 1:** Basic physical properties of the loess.

Soil Sample	Density *ρ* (g/cm^3^)	Dry Density *ρ_d_* (g/cm^3^)	Moisture Content *ω* (%)	Plastic Limit *ω_p_* (%)	Liquid Limit *ω_L_* (%)	Optimum Moisture Content *ω_op_* (%)	Maximum Dry Density *ρ_dmax_* (g/cm^3^)
Loess	1.78	1.63	9.5	9	25	11.2	1.882

**Table 2:** Test program for one-dimensional compression of remolded soil.

Moisture Content (%)	Compaction Degree (%)	Sample Size	Compaction Method	Number of Samples	Axial Pressure (kPa)
7.2	90	60 cm^3^ ring knife sample	Standard proctor compaction	2	25-50-100-200-300-400
	93			2	
	95			2	
	98			2	
9.2	90	60 cm^3^ ring knife sample	Standard proctor	2	25-50-100-200-300-400
	93			2	
	95			2	
	98			2	
11.2	90	60 cm^3^ ring knife sample	Standard proctor	2	25-50-100-200-300-400
	93			2	
	95			2	
	98			2	
13.2	90	60 cm^3^ ring knife sample	Standard proctor	2	25-50-100-200-300-400
	93			2	
	95			2	
	98			2	
15.2	90	60 cm^3^ ring knife sample	Standard proctor	2	25-50-100-200-300-400
	93			2	
	95			2	
	98			2	

## Data Availability

The original contributions presented in this study are included in the article. Further inquiries can be directed to the corresponding author.
